# Dietary Glycotoxins, Advanced Glycation End Products, Inhibit Cell Proliferation and Progesterone Secretion in Ovarian Granulosa Cells and Mimic PCOS-Like Symptoms

**DOI:** 10.3390/biom9080327

**Published:** 2019-07-31

**Authors:** Po-Han Lin, Chih-Chao Chang, Kun-Hsuan Wu, Chun-Kuang Shih, Wenchang Chiang, Hsin-Yuan Chen, Yin-Hwa Shih, Kei-Lee Wang, Yong-Han Hong, Tzong-Ming Shieh, Shih-Min Hsia

**Affiliations:** 1 School of Nutrition and Health Sciences, College of Nutrition, Taipei Medical University, Taipei 11031, Taiwan; 2 Institute of Food Science and Technology, National Taiwan University, Taipei 10617, Taiwan; 3 Department of Healthcare Administration, Asia University, Taichung 41354, Taiwan; 4 Department of Nursing, Ching Kuo Institute of Management and Health, Keelung 20301, Taiwan; 5 Department of Nutrition, I-Shou University, Kaohsiung 84001, Taiwan; 6 School of Dentistry, College of Dentistry, China Medical University, Taichung 40402, Taiwan; 7 Graduate Institute of Metabolism and Obesity Sciences, College of Nutrition, Taipei Medical University, Taipei 11031, Taiwan; 8 School of Food and Safety, Taipei Medical University, Taipei 11031, Taiwan; 9 Nutrition Research Center, Taipei Medical University Hospital, Taipei 11031, Taiwan

**Keywords:** Polycystic ovary syndrome (PCOS), ovarian granulosa cells, advanced glycation end products (AGEs), dehydroepiandrosterone (DHEA), hyperandrogenism

## Abstract

Women with polycystic ovary syndrome (PCOS) have been reported to have an elevated serum advanced glycation end product (AGE) level. However, the effect of AGEs on the pathophysiological ovarian granulosa cells of PCOS is still unclear. In this study, five indented BSA-derived AGE products were used to evaluate their effect on the function of human granulosa cells. We found that the proliferation of both primary human ovarian granulosa (hGC) cells and human granulosa-like tumor (KGN) cells were inhibited by treatment with these five AGE products. The progesterone secretion level was also reduced in both hGC and KGN cells by treatment with these AGE products through downregulation of LH receptor/cAMP regulatory activity. The granulosa cell layer and serum progesterone level were reduced in rats by treatment with MG-BSA; moreover, an increased number of follicle cysts and an irregular estrous cycle were observed. MG-BSA treatment had a similar effect on the phenotypes of the DHEA-induced PCOS model. Additionally, the insulin resistance and hepatic lesions seen in the DHEA-induced PCOS model were observed in the MG-BSA treatment group. Taken together, we found that AGEs exert a toxic effect on ovarian granulosa cells, ovarian morphology, and the estrous cycle that mimics the DHEA-induced PCOS phenotypes.

## 1. Introduction

Polycystic ovary syndrome (PCOS) is one of the most common female endocrine disorders, leading to several complications [1]. The incidence rate of PCOS is up to approximately one in five women of reproductive age [2,3]. Its cardinal features are menstrual cycle irregularities, clinical and biochemical findings of androgen excess, and polycystic ovaries on ultrasound examination. Elevated circulating adrenal precursor androgens, primary dehydroepiandrosterone (DHEA) and dehydroepiandrosterone-sulfate (DHEA-S), are observed in nearly 20–30% of PCOS patients [4,5]. The presence of hyperandrogenism is positively correlated with ovarian dysfunction (anovulation and/or polycystic ovaries) in PCOS patients [6,7], and this phenotype is also observed in DHEA-induced PCOS animal models [8,9]. The diagnosis of PCOS has also been linked to metabolic disorders that encompass insulin resistance, type II diabetes, steatosis, and nonalcoholic steatohepatitis [10,11]. Abnormal glucose tolerance and insulin activation were observed in PCOS patients with hyperandrogenism [12]. A previous study reported that local excessive androgen levels from the follicular fluid are also positively correlated with insulin resistance in PCOS patients, despite normal circulating androgen levels [13]. The pathophysiology of PCOS remains unclear, but there is emerging evidence suggesting that it is a complex trait arising from familial heritable influences, intra- and extrauterine environmental factors, variations in insulin resistance, alterations in steroidogenesis/steroid metabolism, and alternative adaptations to energy excess [14,15].

Recently, some dietary ingredients have been noticed to increase the risk of PCOS. Advanced glycation end products (AGEs), also known as glycotoxins, naturally exist in uncooked animal foods. However, thermal processing and modifications can lead to increased formation of AGEs, especially long-term or high-temperature cooking methods such as roasting, grilling, and frying. For example, the amount of AGEs in steaks and roast chickens is much higher than in other foods [16]. AGEs are produced by glycation. The glycation reaction is also called the Maillard reaction [17]. The Maillard reaction is a reaction between the carbonyl group of reducing sugar and the amine group of amino acids. Due to the formation of dark brown melanoidins at the end of the reaction, it is also referred to as nonenzymatic browning [18]. AGEs are a complex group of molecules that have been found almost everywhere in tissues and organs of the human body [19]. The source of human AGEs can be divided into endogenous and exogenous sources. Endogenous sources are self-synthesized in the body under normal physiological conditions and gradually accumulate with age. Exogenous AGEs are mainly ingested and smoked by cigarettes [20]. Excessive AGEs in the body are known to have a negative impact on the human body, possibly through an increase in oxidative stress, irreversible cross-linking with protein in the body, affecting signal transduction, and altering protein configurations [21,22,23,24].

It has been suggested that AGEs are associated with the development of chronic diseases, such as type II diabetes, obesity, metabolic syndrome, and other conditions [25,26,27]. In PCOS, an elevated serum AGE level has been observed and associated with the pathogenesis of insulin resistance [28,29]. However, the effects of AGEs on ovarian function and hormone regulation, which are critical features of PCOS, are still unclear. Herein, we investigated the effect of AGEs on ovarian granulosa cell growth and hormone secretion; moreover, a DHEA-induced PCOS animal model was generated for evaluation of the role of AGEs on PCOS.

## 2. Materials and Methods

### 2.1. AGEs (GA-BSA, GO-BSA, GOA-BSA, GLU-BSA, and MG-BSA) Preparation

Bovine serum albumin (BSA), glyoxal (GO), glycolaldehyde (GOA), glucose (GLU), methylglyoxal (MG), and glyceraldehyde (GA) were purchased from Sigma-Aldrich Chemical Co. (St. Louis, MO, USA). These AGE precursors, GO (0.1 M), GOA (0.1 M), GA (0.1 M), MG (0.1 M), and GLU (0.5 M), were prepared as solutions in phosphate buffer (0.2 M potassium dihydrogen phosphate and 0.2 M potassium hydrogen phosphate) with BSA (25 mg/mL) under sterile condition. The solutions were incubated at 37 °C for 14 days and then concentrated through a dialysis membrane. After completion, dialysis was performed again and the protein concentration was quantified. Nonglycated BSA as a control was prepared under the same conditions. Over 99% of endotoxins were removed using resins (EndotoxinOUT Resin; G-Bioscience, St. Louis, MO, USA). The endotoxin concentration in AGEs was determined using endotoxin analysis reagents (ToxinSensor Chromogenic LAL Endotoxin Assay; GenScript, Piscataway, NJ, USA). Solutions with an endotoxin concentration of <0.7 EU/mL were used as AGEs. These AGE products were named: GA-BSA, GO-BSA, GOA-BSA, GLU-BSA, and MG-BSA in this study. AGE activity was assessed by fluorescence at excitation/emission wavelengths of 340/410 nm. The fluorescence of these five AGE products was 4.5–26.5-fold stronger than that of control nonglycated BSA (Supplementary Figure S1).

### 2.2. Cell Culture Conditions

Human follicular fluid can provide a source of human granulosa cells (hGC) for medical study. Ovarian granulosa cells were collected from a donor who underwent in vitro fertilization (IVF) at the Fertility and Reproductive Medicine Center of National Taiwan University Hospital (NTUH; Taipei, Taiwan). In this study, all human specimens were approved by the Institutional Review Board and Ethics Committee of National Taiwan University Hospital (Permit Number: 201301013RINC). Collection of follicular fluid and culture of primary ovarian granulosa cells were performed according to the method of a previous report by Quin and colleagues [30]. Briefly, the 50% Percoll gradient solution was used to remove red blood cells and the aggregated layer of granulosa cells was harvested, washed, and processed for further in vitro studies. The primary ovarian granulosa cells were maintained in DMEM-F12 medium (Gibco, Grand Island, NY, USA) and supplemented with 10% fetal bovine serum (FBS; Gibco) and 1% antibiotics (100 units/mL penicillin, 0.1 μg/mL streptomycin, 0.25 μg/mL amphotericin) in a humidified atmosphere of 5% CO_2_ at 37 °C.

A human granulosa-like tumor cell line (KGN) was purchased from RIKEN Bioresource Research Center (RCB1154; Ibaraki, Japan). KGN cells were also maintained in DMEM-F12 medium (Gibco) containing 10% FBS and 1% antibiotics as the above description.

### 2.3. Immunofluorescence Staining

It is known that the follicle-stimulating hormone (FSH) receptor is abundantly expressed in ovarian granulosa cells [31]. Briefly, at the end of incubation, the hGC cells (5 × 10^5^ cells per well) were fixed in methanol for 30 min at 4 °C, and then incubated with 5 μg/mL FSH receptor antibody (Abcam, Cambridge, MA, USA) for 1 h at room temperature. The fluorescent secondary antibody was prepared in blocking buffer, and subsequently incubated with the cells for an additional 60 min at room temperature.

In addition, KCN cells were mounted on coverslips, washed with PBS, fixed with 4% paraformaldehyde for 15 min at 37 °C, permeabilized with 0.5% (*v*/*v*) Triton X-100 for 20 min, and blocked with 1% (*w*/*v*) goat serum albumin for 30 min. Samples were then washed, probed at 4 °C overnight with an antibody against receptor of advanced glycation end product (RAGE; Millipore, Burlington, MA, USA), and then incubated with a secondary antibody for 40 min. After washing with PBS, the cells were mounted with DAPI (2-(4-amidinophenyl)-6-indolecarbamidine dihydrochloride)-containing mounting medium for fluorescence (Vector Laboratories, Burlingame, CA, USA) and analyzed under a fluorescence microscope (EVOS^®^ microscope; Thermo Fisher Scientific, CA, USA).

### 2.4. Cell Proliferation Analysis

The MTT (3-(4,5-dimethylthiazol-2-yl)-2,5-diphenyltetrazolium bromide; Sigma-Aldrich) assay was performed as previously described [32]. KGN cells and hGC cells (5000 cells/well) were seeded into 96-well plates. After the cells attached, they were treated with the five AGE products for four and eight days. After the incubation period, 0.5 mg/mL of the MTT reagent was added and incubated at 37 °C for 3 h. Subsequently, the media were removed and crystal formazan was dissolved using DMSO (Sigma-Aldrich). The optical density was measured using the Epoch Microplate Spectrophotometer (BioTek, Winooski, VT, USA) at 570 nm and 630 nm as the reference wavelength. The relative cell viability is presented as a percentage of cells treated with vehicle.

### 2.5. Western Blot Analysis

Cells were lysed in RIPA buffer containing a protease inhibitor mixture and a phosphatase inhibitor cocktail tablet (Roche Diagnostics, Basel, Switzerland). The protein concentration was analyzed by the bicinchoninic acid (BCA) assay kit (T-Pro Biotechnology, New Taipei City, Taiwan). Quantified protein sample (20 μg) was resolved on 7.5–15% sodium dodecyl sulfate polyacrylamide gel electrophoresis (SDS-PAGE), and then proteins were transferred from the gel to polyvinylidene difluoride (PVDF) membrane. The membrane was then blocked in 5% BSA solution for 1 h and incubated with the anti-RAGE (1:1000; Millipore) and PCNA (1:1000; Cell Signaling) antibodies overnight at 4 °C. The next day, the membrane was incubated with secondary anti-rabbit or anti-mouse antibodies (1:10,000; Cell Signaling) for 1 h at room temperature. The membrane was reacted with the Electrochemiluminescence (ECL) reagent and the visual signals were detected using a Luminescent Image Analyzer Amersham Imager 600 (GE Healthcare Life Sciences, MA, USA). The band densities were quantified using the Image-J software program.

### 2.6. Measurement of Progesterone Release from KGN Cells

KGN cells (10^5^ cells per well) were seeded into 24-well plates. After the cells attached, they were treated with AGE products alone or in combination with hCG (0.5 IU/mL), forskolin (10^−5^ M), or 8-Br-cAMP (10^−4^ M) for 24 h. The culture media were collected and the progesterone concentration was detected using a commercial Cayman progesterone enzyme immunoassay kit (Cayman Chemical, Ann Arbor, MI, USA). All procedures were performed according to the standard manufacturer’s protocols.

### 2.7. Animals

Three-week-old female Sprague–Dawley (SD) rats were purchased from BioLASCO Taiwan Corporation (Taipei, Taiwan). All animal experimental procedures in this study were approved by the Institutional Animal Care and Use Committee (IACUC), Taipei Medical University (Permit number: LAC-2013-0292). Rats were housed in an environment with a constant humidity and temperature (24 ± 2 °C; 50–60% humidity) and a 12 h light/dark artificial illumination cycle (0700-1900). During the experiment, the animals had free water access and were given a chow diet. Rats were divided into four groups according to random sampling. After one week of adaptation, administration and oral gavage were performed. In this study, the animals’ growth weight was recorded every three days. The intervention of methylglyoxal glycation end products (MG-AGEs) was administered daily by oral gavage at 40 mg/kg body weight [33].

### 2.8. Dehydroepiandrosterone (DHEA)-Induced Polycystic Ovarian Syndrome (PCOS) Animal Model

A DHEA-induced hyperandrogenemia-like animal model has been widely used to study PCOS [34,35]. A diagram of the dehydroepiandrosterone (DHEA)-induced PCOS rat model is illustrated in Figure 5A. DHEA was dissolved in sesame oil and given to the rats by subcutaneous injection (6 mg/100 g body weight). The daily treatments lasted for up to 37 days consecutively. Thereafter, estrous cycles of all rats were determined by analyzing the cell types in vaginal smears for an additional 1 week.

### 2.9. Measurement of Serum Hormones and Biochemical Indexes

Fasting blood glucose levels were measured using a commercially available blood glucose meter (OK-1B; OK Biotech, Hsinchu, Taiwan). Serum androstenedione concentration was measured using a commercially available IBL-America androstenedione enzyme immunoassay kit (IBL International GmbH, Hamburg, Germany). Serum estradiol and progesterone levels were measured using a commercial Cayman enzyme immunoassay kit (Cayman Chemical, Ann Arbor, MI, USA). Serum insulin level was measured using the Mercodia ultrasensitive rat insulin ELISA kit (Mercodia AB, Uppsala, Sweden). The liver indexes AST and ALT were measured with spotchemTM II reagent strips using a SpotchemTM EZ automated dry chemistry analyzer (Arkray Global Business Inc., Kyoto, Japan). All procedures were performed according to the standard manufacturer’s protocols.

### 2.10. Statistical Analysis

The data are presented as the means ± standard deviations (SD). The experimental data was statistically analyzed by the Student *t*-test or one-way ANOVA using SPSS computer software and Duncan’s multiple test was used for postmortem testing. Statistical significance was reached when the *p* value < 0.05.

## 3. Results

### 3.1. Effects of AGE Products on the Cell Proliferation of Human Granulosa Cells (hGC) and Human Granulosa-Like (KGN) Tumor Cells

**Figure 1:**
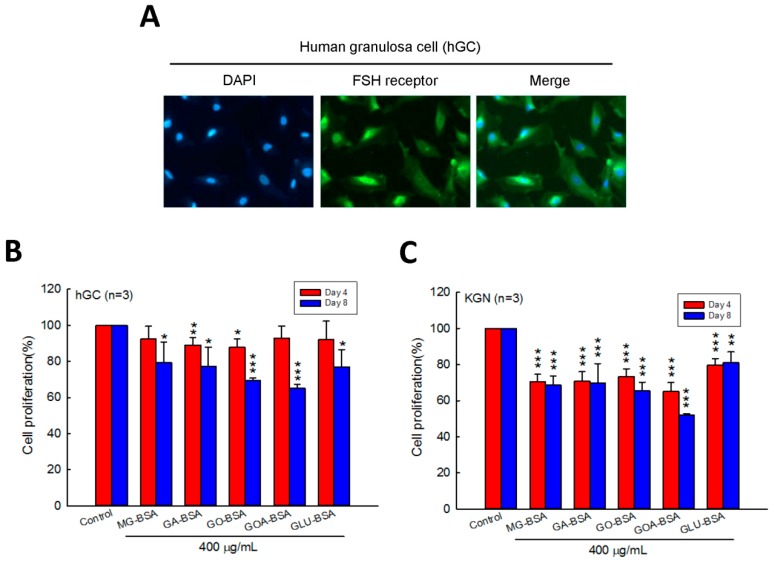
Effects of AGEs on the cell proliferation of human granulosa cells (hGC) and human granulosa-like (KGN) tumor cells. (**A**) Fluorescence microscopy analysis of the expressions of the FSH receptor (green) in human ovarian granulosa cells. DAPI (blue) was used to counterstain the nuclei. Overlay is presented. Treatment of both (**B**) hGC (5 × 10^3^ cells per well) and (**C**) KGN (5 × 10^3^ cells per well) cells with BSA (400 μg/mL) as a control or five different AGEs for four and eight days. The cell proliferation rate was analyzed using the MTT assay. Data are shown as the mean ± SD (*n* = 3). * *p* < 0.05, ** *p* < 0.01, *** *p* < 0.001 compared with the control group. MG-BSA, methylglyoxal- bovine serum albumin; GA-BSA, glyceraldehyde-bovine serum albumin; GO-BSA, glyoxal-bovine serum albumin; GOA-BSA, glycolaldehyde-bovine serum albumin; GLU-BSA, glucose-bovine serum albumin.

Human primary ovarian granulosa cells (hGC) were purified from the donor who underwent in vitro fertilization (IVF). Immunofluorescence staining of the FSH receptor was performed to identify the purity of the hGC cells. The results showed that the FSH receptor was intensively expressed in hGC cells (Figure 1A). To evaluate the role of AGEs in the cell proliferation of granulosa cells, five identified AGE products were used in this study. We found that the cell proliferation of hGC cells was inhibited by treatment with GA-BSA and GO-BSA for four days; however, when treated for eight days, all five AGE products inhibited hGC cell proliferation (Figure 1B). In addition, treatment with the five AGE products for four and eight days reduced cell proliferation of KGC cells (Figure 1C).

**Figure 2:**
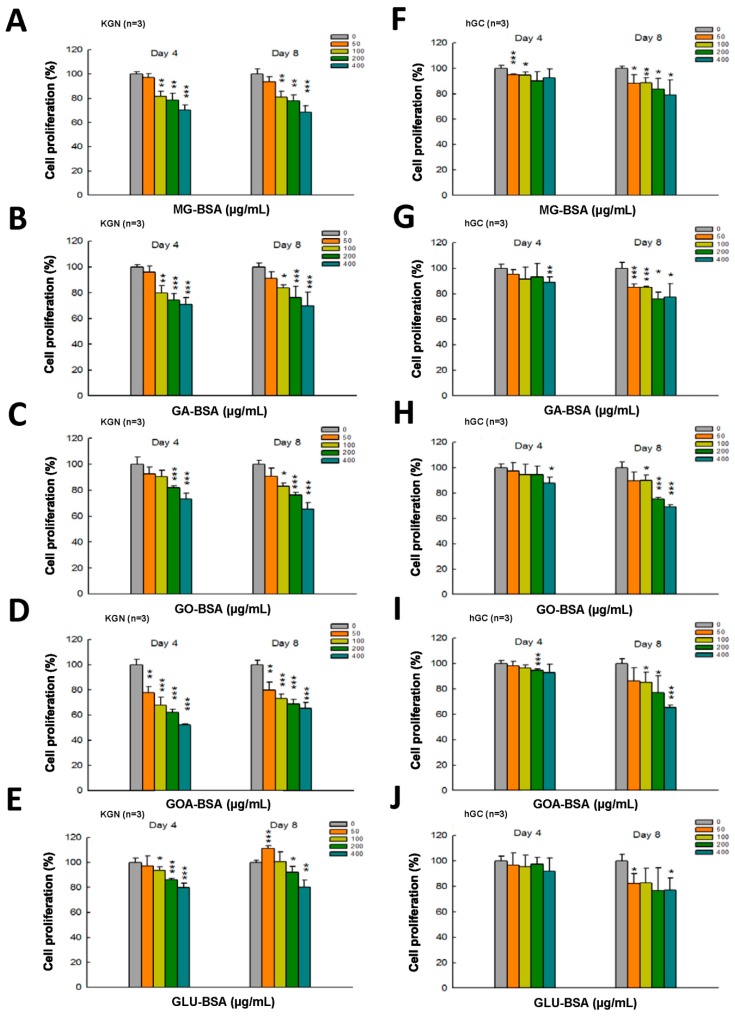
Dose–response assessment of AGE products in KGN and hGC cells. Both KGN and hGC cells were seeded into a 96-well plate (5 × 10^3^ cells per well). After the cells attached on the bottom of the plate, they were then treated with BSA (400 μg/mL) as a control and (**A**, **F**) MG-BSA, (**B**,**G**) GA-BSA, (**C**,**H**) GO-BSA, (**D**,**I**) GOA-BSA, and (**E,J**) GLU-BSA for four and eight days, respectively. Data are shown as the mean ± SD (*n* = 3). * *p*< 0.05, ** *p* < 0.01, ***p < 0.001 compared with the control group.

The dose–response assessment of AGE products was further performed in both KGN and hGC cells. The results showed that treatment with all five individual AGE products in serial doses for four and eight days inhibited the proliferation of KGN cells (Figure 2A–D). A slight inhibitory effect on the proliferation of hGC by AGE products was observed at day 4 of the treatment (Figure 2E–I), but the proliferation of hGC cells was significantly reduced at day 8 of the AGE products treatment (Figure 2E–I).

### 3.2. MG-BSA-Induced RAGE Expression in KGN Cells

**Figure 3:**
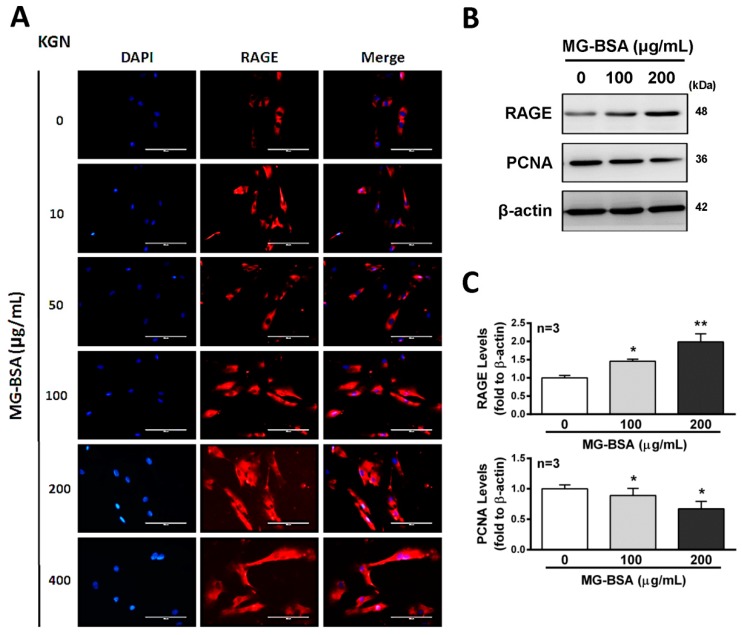
Increasing levels of RAGE in KGN cells treated with MG-BSA. KGN cells were treated with MG-BSA in serial doses for 48 hr. (**A**) Fluorescence microscopy analysis of the expressions of RAGE (red) in KGN cells. DAPI (blue) was used to counterstain the nuclei. The overlay is presented. Scale bar = 100 μm. (**B**) Western blotting was used to analyze the expression of RAGE and PCNA in KGN cells with MG-BSA treatment for 48 hr. (**C**) Each target protein was normalized to β-actin expression. Data are shown as the mean ± SD (*n* = 3). *p < 0.05, **p < 0.01 compared with the control group.

Activation of the receptor for advanced glycation end products (RAGE) protein expression has been reported to stimulate cell death signaling activation [36], whereas some reports indicated the opposite [37]. Therefore, we further examined the role of RAGE in reducing cell proliferation by treatment with AGE products. The results showed that treatment of KGN cells with MG-BSA for 48 h increased the expression of RAGE in a dose-dependent manner (Figure 3A–C). Meanwhile, the expression of PCNA, a biomarker of proliferation, was reduced by treatment with MG-BSA for 48 h (Figure 3B,C).

### 3.3. Effect of AGE Products on the Secretion of Progesterone in KGN Cells

**Figure 4:**
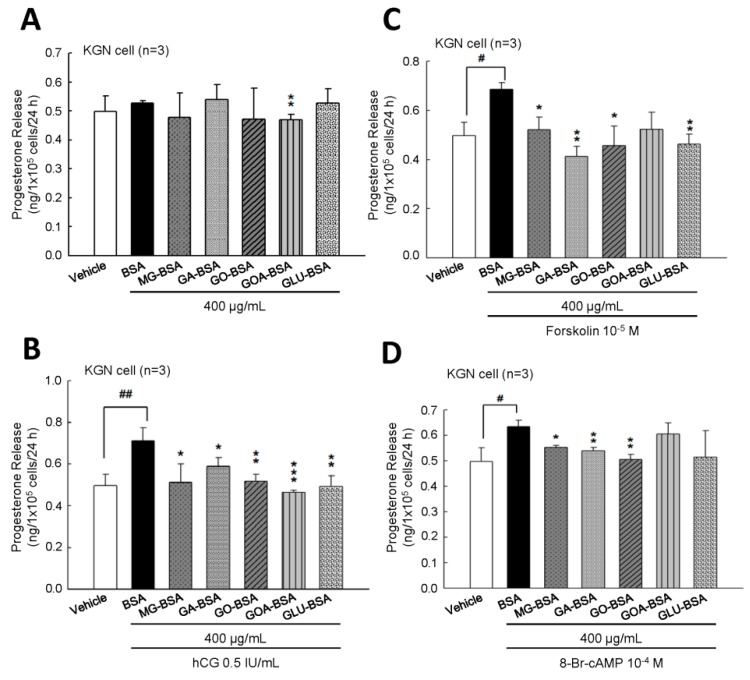
The inhibitory effect of AGE products on the progesterone release from KGN cells. (**A**) After treatment of BSA (400 μg/mL) as a control and AGE products (400 μg/mL) for 24 h, the media were collected and then the level of progesterone was measured using ELISA. KGN cells were treated with AGE products in the presence of (**B**) hCG (0.5 IU/mL), (**C**) forskolin (10^−5^ M), and (**D**) 8-Br-cAMP (10^−4^ M) for 24 h. Data are shown as the mean ± SD (*n* = 3). * *p* < 0.05, ** *p* < 0.01 compared with the control group; ^#^ *p*< 0.05, ^##^ *p* < 0.01 compared with the vehicle group.

To evaluate whether the biological function of KGN cells was inflected by treatment with AGE product, the secreted progesterone concentration was measured. The results showed that treatment of five individual AGE products of 400 μg/mL for 24 h slightly reduced progesterone secretion from KGN cells (Figure 4A). However, when cotreated with hCG, as an analog of LH, these five AGE products all inhibited the hCG-evoked progesterone level (Figure 4B). We further investigated whether the AGE interfered with the activity of the LH-regulated downstream molecule, cAMP. The results showed that the forskolin (an activator of adenylyl cyclase)- or 8-Br-cAMP (a permeable analog of cAMP)-stimulated progesterone secretion from KGN cells was reduced in the presence of AGE products (Figure 4B,C). In order to demonstrate that this inhibitory effect did not cause cell death, the MTT assay was used to measure the cell proliferation after treatment with AGE products for 24 h. The results showed that cell proliferation of KGN cells was not affected by treatment with these five AGE products at 400 μg/mL for 24 h (Supplementary Figure S2).

### 3.4. Effects of MG-BSA on the DHEA-induced PCOS Rat Model

**Figure 5:**
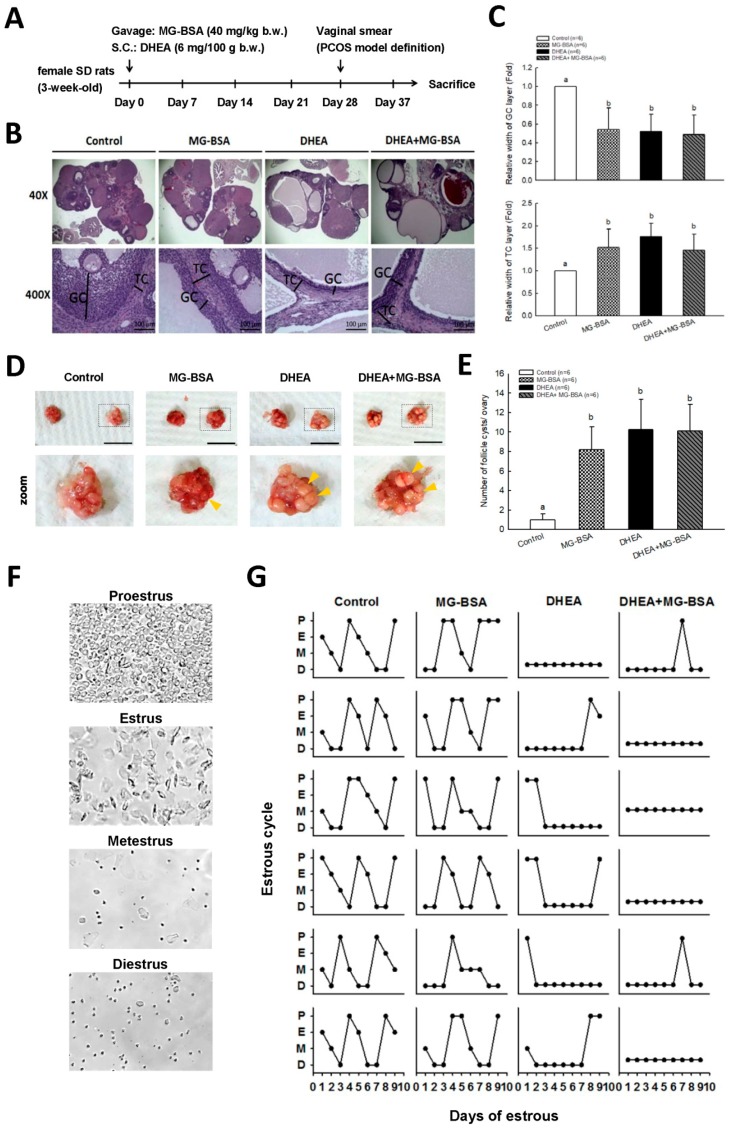
Effects of dietary MG-BSA on DHEA-induced PCOS rats. (**A**) Graphical depiction of the PCOS model via DHEA induction and daily MG-BSA treatment. After treatment for four weeks, the estrous cycle was monitored by vaginal smears for an additional nine days. (**B**) Ovarian sections were stained with hematoxylin and eosin (H&E) to evaluate the histopathologic changes. Scale bar = 1 μm. GC, granulosa cells; TC, theca cells. (**C**) Quantitative analysis of the width of the GC and TC area in a cross-section. (**D**) The bilateral ovaries were photographed. The yellow arrow indicates the site of follicle cysts. Scale bar = 1 cm. (**E**) The number of follicle cysts was counted. (**F**) The typical cell type of the estrous cycle was photographed. P, proestrus; E, estrus; M, metestrus; D, diestrus. (**G**) Nine continuous days of the estrous cycle were monitored by vaginal smear. Data are represented as mean ± SD. Different letters indicate significant differences among all groups (*p* < 0.05).

The rat in vivo model was created as shown in Figure 5A. Rats were subcutaneously injected with DHEA (6 mg/100 g) and administered MG-BSA (40 mg/kg) by oral gavage for 37 days. At the end of the experiment, the ovaries were isolated and photographed. Histopathological analysis was performed on the ovarian tissues. The hematoxylin and eosin H&E stain results showed that treatment of rats with MG-BSA reduced granulosa cell (GC) layer compared to the control group, and similar results were observed in the DHEA-induced PCOS group (Figure 5B,C). However, this reduced effect was not worsened by treatment with MG-BSA in DHEA-induced PCOS rats (DHEA + MG-BSA) (Figure 5B,C: top panel). Conversely, the opposite was observed for the theca cell (TC) layer (Figure 5C: bottom panel). The morphology of the ovaries was assessed after sacrifice. The number of follicle cysts was higher in the MG-BSA-treated group, DHEA group, and DHEA + MG-BSA group (Figure 5D,E). After treatment for four weeks, a vaginal smear was performed to evaluate the effect of MG-BSA on the estrous cycle. The results showed that an irregular estrous cycle was observed in the MG-BSA-treated group. This irregular estrous cycle was also observed in the DHEA-induced PCOS group (Figure 5F,G). However, the number of non-estrous cycle rats was higher in the DHEA + MG-BSA groups (four of six rats) compared to DHEA (two of six rats) (Table 1).

**Figure 6:**
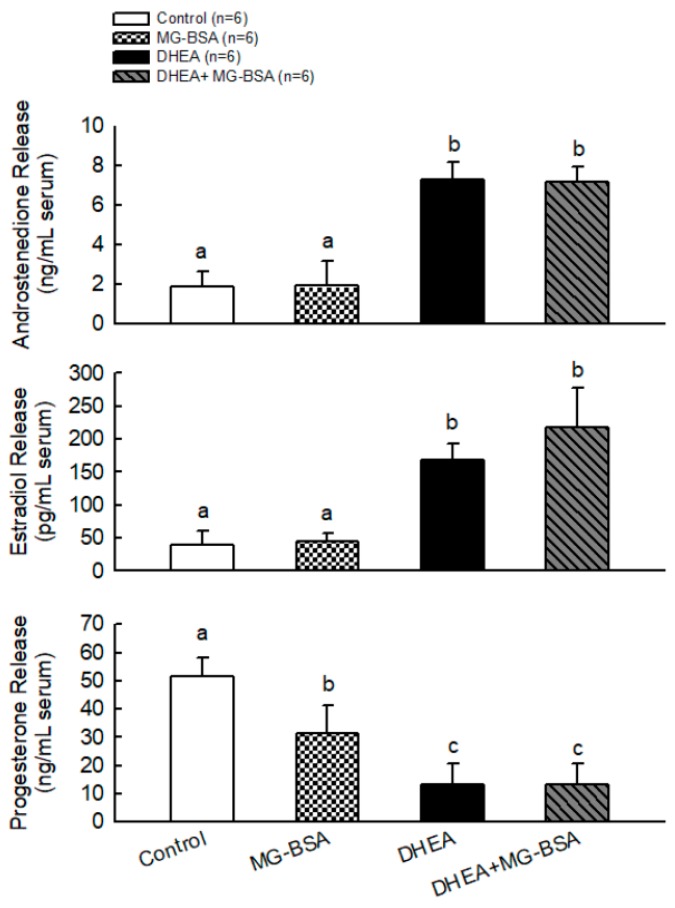
Effects of MG-BSA on serum hormone (androstendione, E2, and P4) levels of DHEA-induced PCOS rats. After sacrifice, blood was collected and serum was isolated. The serum levels of (**A**) androstendione, (**B**) estradiol, and (**C**) progesterone were measured using ELISA. Data are represented as mean ± SD (*n* = 8). Different letters indicate significant differences among all groups (*p* < 0.05).

To further understand whether MG-BSA-induced ovarian deterioration caused its physiological dysfunction, the serum androstenedione, estradiol, and progesterone concentrations were measured. The results showed that MG-BSA treatment did not alter the serum levels of androstenedione and estradiol compared with the control group, but treatment with MG-BSA reduced serum progesterone levels (Figure 6). Moreover, the DHEA-induced abnormal levels of androstenedione, estradiol, and progesterone were not altered by MG-BSA treatment (Figure 6).

### 3.5. Effect of MG-BSA on Glucose Homeostasis and Liver Function Index

**Figure 7:**
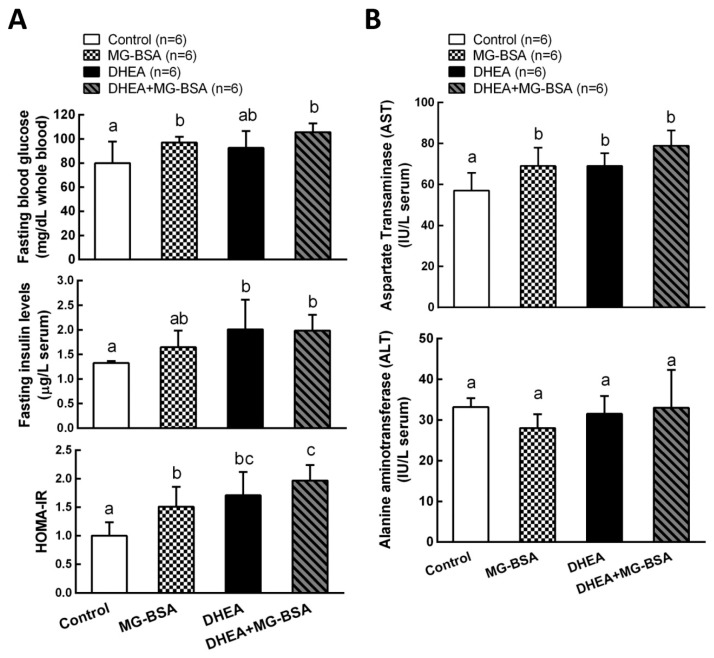
Effects of MG-BSA on glucose homeostasis and the liver functional index in DHEA-induced PCOS rats. Before sacrifice, rats were starved for 12 h. Blood was collected and then serum was isolated. (**A**) Serum glucose and insulin levels were measured. The homeostatic model assessment–insulin resistance (HOMA-IR) index was calculated via the formula: HOMA-IR = fasting insulin × fasting glucose/405. (**B**) Serum AST and ALT levels were measured to evaluate the index of liver function. Data are represented as mean ± SD (*n* = 8). Different letters indicate significant differences among all groups (*p* < 0.05).

It has been reported that the hyperandrogenism of PCOS is positively correlated with the development of insulin resistance [13,38]. Therefore, we further investigated the role of AGE in the pathogenesis of the DHEA-induced PCOS model. The results showed that fasting blood glucose was higher after treatment with MG-BSA compared to the control group. This increase was also observed in the DHEA-induced PCOS group, but it was not altered by DHEA + MG-BSA (Figure 7A, top panel). The serum insulin level was not affected by treatment with MG-BSA (Figure 7B, middle panel). According to the homeostatic model assessment–insulin resistance (HOMA-IR) index results, treatment with MG-BSA alone or combined DHEA with MG-BSA (DHEA + MG-BSA) increased the risk of insulin resistance (Figure 7A, bottom panel). Additionally, it has been reported that PCOS patients have a high risk of liver dysfunction [39,40]. Hence, the effect of AGE on the liver functions was also evaluated in this study. The results showed that the hepatic aspartate aminotransferases (AST) level increased after treatment with MG-BSA. This enhanced effect was also observed in DHEA-induced PCOS groups, but it was not altered by DHEA + MG-BSA (Figure 7B, top panel). However, the alanine aminotransferases (ALT) level in the liver was not affected (Figure 7B, bottom panel).

## 4. Discussion

PCOS is characterized by excess androgen that causes the pathogenesis of folliculogenesis and failure in dominant follicle selection [41]. The hyperandrogenism is related to the ovarian dysfunction, which is one of the trials of infertility in PCOS [42]. It has been reported that accumulation of AGE by treatment of mice with D-galactose exerts PCOS-like phenotype, especially on hormonal imbalances and granulosa cell dysfunction [43]. A few reports have shown that the elevated serum AGE level was observed in PCOS patients [28,29]. In the present study, our results showed that AGEs inhibited the proliferation of the primary human ovarian granulosa (hGC) cells and human granulosa-like tumor (KGN) cells. The secretion of progesterone from both cells was reduced by treatment with AGEs. We also found that rats treated with AGEs exerted several phenotypes similar to the DHEA-induced PCOS model.

AGEs have been reported to affect cell growth. A previous study demonstrated that AGEs suppress islet vascular endothelial cell growth through activation of the apoptosis pathway [44]. In this study, our results showed that treatment with AGEs inhibited the proliferation of granulosa cells and smaller GC layers were observed in the animal model. Treatment with MG-BSA increased RAGE protein expression in KGN cells, whereas downregulation of PCNA expression was observed, suggesting that RAGE could be involved in this regulatory pathway. DNA-damage-induced proliferating cell nuclear antigen (PCNA) ubiquitination serves as the key event mediating postreplication repair [45]. However, AGEs binding to their membrane-bound receptor RAGE are, in turn, involved in the regulation of cell growth via several pathways [46]. Therefore, the precise mechanism needs to be investigated further.

The levels of MG-BSA in serum and ovarian tissues were measured in this study. Interestingly, the result showed that the serum level of MG-BSA was lower in the MG-BSA group compared with the control group, and a similar pattern was observed in the DHEA treatment and DHEA + MG-BSA groups (Supplementary Figure S3A). However, the MG-BSA level was not altered in the ovarian tissues (Supplementary Figure S3B). The trend of MG-BSA level from these results was not incompatible with the treatment of rats with MG-BSA. A previous study has reported that after intravenous injection with MG-BSA into circulation, MG-BSA was rapidly accumulated in the liver and kidney compared with the BSA group [47]. The clearance rates for MG-BSA in the liver and kidney were also larger than those for BSA [47]. These results might explain the lower level of MG-BSA in the circulation after MG-BSA treatment.

Human chorionic gonadotropin (hCG) has high homogeneity and can bind to luteinizing hormone receptor to stimulate the production of steroid hormones [48]. Our results showed that progesterone secretion was increased by 0.5 IU/mL with hCG treatment; however, treatment with all five AGE products inhibited the hCG-evoked progesterone secretion in KGN cells. In addition, treatment of KGN cells with the AGE products reduced forskolin- and 8-Br-cAMP-stimulated progesterone secretion. These results suggest that AGE reduced progesterone secretion through impairment of LH receptor-regulated adenylyl cyclase activity.

The DHEA-induced PCOS rat model impairs ovarian function and induces morphological changes [49,50]. Consistently, our results revealed that DHEA-induced PCOS rats exhibited a smaller GC layer, whereas the TC cell layer increased, and an increased number of follicle cysts were observed. Interestingly, we observed these phenotypes in the ovaries of rats treated with MG-BSA as mentioned above, but MG-BSA treatment did not aggravate these effects on the ovaries of DHEA-induced PCOS rats. Despite the progesterone level being reduced by MG-BSA treatment, the serum levels of DHEA and estradiol were not altered by treatment with MG-BSA in normal and DHEA-induced PCOS rats. These results suggest that treatment with MS-BSA could exhibit PCOS-like phenotypes. Moreover, we found that the estrous cycle became irregular with MG-BSA treatment, which was aggravated in DHEA-induced PCOS rats who were administered MG-BSA, where the rate of nonestrous cycle was increased.

It is well understood that estrogen is mainly produced by the granulosa cells. According to this study, MG-BSA reduced granulosa cell proliferation and the granulosa cell layer was reduced in both normal and PCOS model groups, but the serum estradiol level was not altered. Therefore, whether the peripheral tissues, such as adipose tissue and the liver, keep aromatase activation to maintain the level of estradiol will be an interesting problem for further study. In addition, patients with PCOS have high serum levels of androgen and estrogen, leading to failure of ovulation. Therefore, a low level of progesterone in patients with PCOS has been observed [51].

The homeostatic model assessment–insulin resistance (HOMA-IR) index was widely used to estimate the insulin sensitivity [52,53]. Approximately 20–40% of PCOS patients have been reported to have insulin resistance [54]. According to the HOMA-IR data in this study, high risk of insulin resistance was also observed after treatment with MG-BSA in normal and DHEA-induced PCOS animal models. Previous studies have shown that limiting intake of glycation end products can improve insulin resistance in diabetic patients [55]. It has been reported that AGEs trigger the generation of intracellular reactive oxygen species, which causes an inhibition of glucose uptake [56]. Moreover, AGEs have been reported to interfere with insulin activation in KGN cells through downregulation of PI3K-AKT activity and suppress glucose uptake by inhibition of glucose transporter translation to the cell membrane [57]. Long-term insulin resistance and glucose uptake impairment can cause whole liver lesions, such as nonalcoholic fatty liver disease (NAFLD) [58]. Recently, the correlation between PCOS and NAFLD is considered an important problem and a hotbed for research [59]. In this study, the alanine aminotransferases (ALT) and aspartate aminotransferases (AST) were used to evaluate the level of hepatic injury. The results showed that PCOS increased serum AST levels. MG-BSA also increased serum AST levels, but serum ALT levels were not altered by MG-BSA treatment. These results suggest that AGE treatment could impair hepatic lesions which mimic the PCOS-like phenotype.

## 5. Conclusions

In conclusion, our results showed that AGE products exert a toxicity effect on ovarian granulosa cells, especially on cell proliferation and hormone release. Ovarian morphology and the estrous cycle were affected by MG-BSA, suggesting that all phenotypes exerted from MG-BSA-treated rats mimic the DHEA-induced PCOS group.

## Figures and Tables

**Table 1:** Estrous cycle assessment after MG-BSA treatment was summarized as shown in the table.

Estrous cycle assessment after MG-BSA treatment				
Group	Total No.	No. of Regular Cycle	No. of Irregular Cycle	No. of Non-Cycle
Control	6	6	0	0
MG-BSA	6	2	4	0
DHEA	6	0	4	2
DHEA + MG-BSA	6	0	2	4

## Supplementary Materials

The following are available online at https://www.mdpi.com/2218-273X/9/8/327/s1, Figure S1: The property of AGE products. Figure S2. Effects of AGE products on the cell viability of KGN cells. Figure S3. The MG-BSA level in the serum and ovarian tissues.
