# Supplementary material for: Dietary Glycotoxins, Advanced Glycation End Products, Inhibit Cell Proliferation and Progesterone Secretion in Ovarian Granulosa Cells and Mimic PCOS-Like Symptoms
Source: Biomolecules. 2019 Jul 31;9(8):327. doi: 10.3390/biom9080327 (PMC6723748; doi:10.3390/biom9080327)
Supplement: Supplementary file 1 [file biomolecules-09-00327-s001.pdf]

Supplementary Information

Dietary Glycotoxins, Advanced Glycation End Products, Inhibit Cell Proliferation and Progesterone Secretion in Ovarian Granulosa Cell and Mimic PCOS-like Symptoms

Po-Han Lin [1], Chih-Chao Chang [1], Kun-Hsuan Wu [2], Chun-Kuang Shih [1], Wenchang Chiang [2], Hsin-Yuan Chen [1], Yin-Hwa Shih [3], Kei-Lee Wang [4], Yong-Han Hong [5], Tzong-Ming Shieh [6] and Shih-Min Hsia [1,7,8,9,*]

[1] School of Nutrition and Health Sciences, College of Nutrition, Taipei Medical University, Taipei 11031, Taiwan
[2] Institute of Food Science and Technology, National Taiwan University, Taipei 10617, Taiwan
[3] Department of Healthcare Administration, Asia University, Taichung 41354, Taiwan
[4] Department of Nursing, Ching Kuo Institute of Management and Health, Keelung 20301, Taiwan
[5] Department of Nutrition, I-Shou University, Kaohsiung 84001, Taiwan
[6] School of Dentistry, College of Dentistry, China Medical University, Taichung 40402, Taiwan
[7] Graduate Institute of Metabolism and Obesity Sciences, College of Nutrition, Taipei Medical University, Taipei 11031, Taiwan
[8] School of Food and Safety, Taipei Medical University, Taipei 11031, Taiwan
[9] Nutrition Research Center, Taipei Medical University Hospital, Taipei 11031, Taiwan

* Correspondence: bryanhsia@tmu.edu.tw; Tel.: +886-2-2736-1661 (ext. 6558)

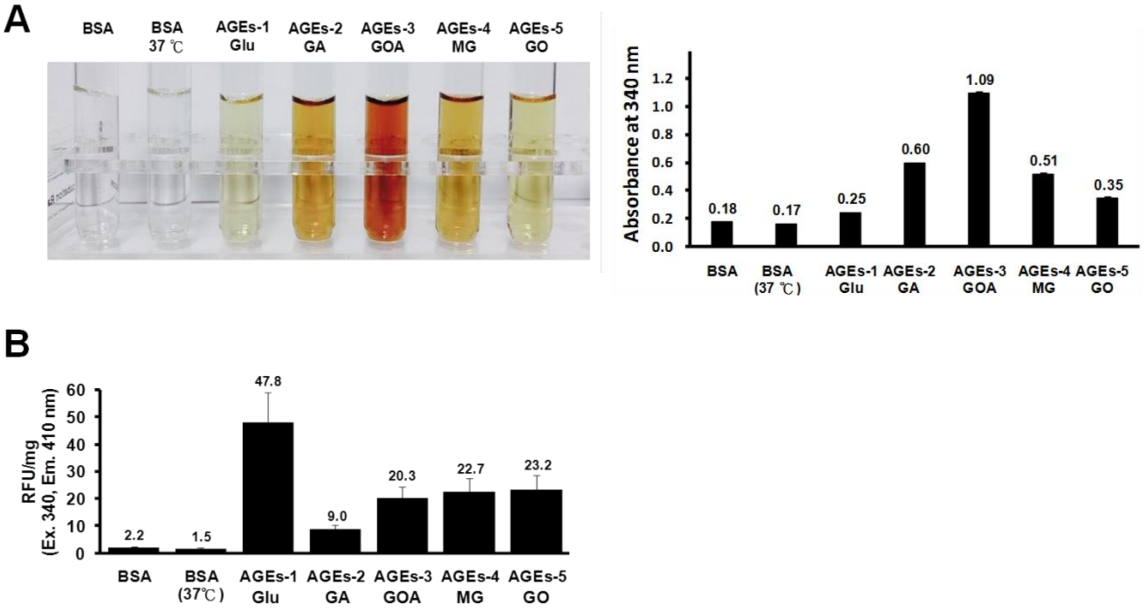

Supplementary Figure S1. The property of AGE products. (A) After incubation at 37°C for 14 days, the color in these five AGE products was photographed and quantified by measuring absorbance at 340 nm. **(B)** These five AGE products activity were also assessed by the fluorescence at excitation/emission wavelengths of 340/410 nm.

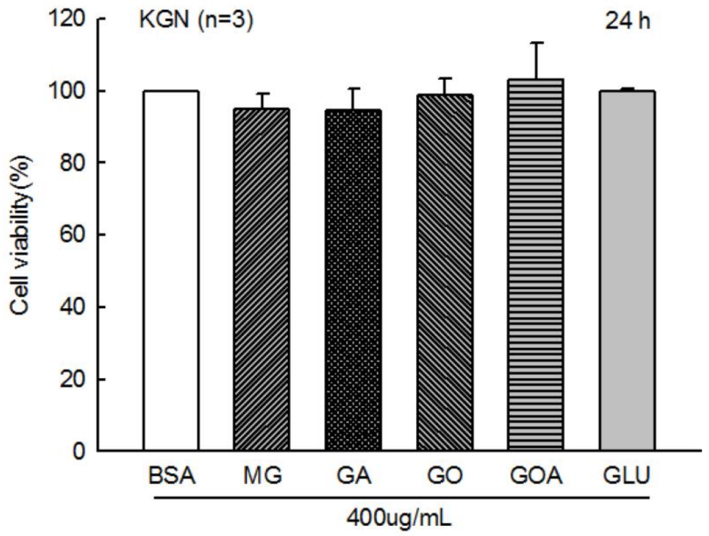

Supplementary Figure S2. Effects of AGE products on the cell viability of KGN cells. KGN cells (5000 cells per well) were seeded into a 96-well plate. After the cells attached on the bottom of plate, they were treated with these five AGE products at 400 μg/mL for 24 h. At the end of incubation, media were removed and then incubated with 0.5mg/ml MTT solution for 3 h. Subsequently, media were removed and crystal formazan was dissolved using DMSO. The optical density was measured using the Epoch Microplate Spectrophotometer (BioTek, Winooski, VT, USA) at 570 nm and 630 nm as the reference wavelength. Data are shown as the mean ± SD (n = 3).

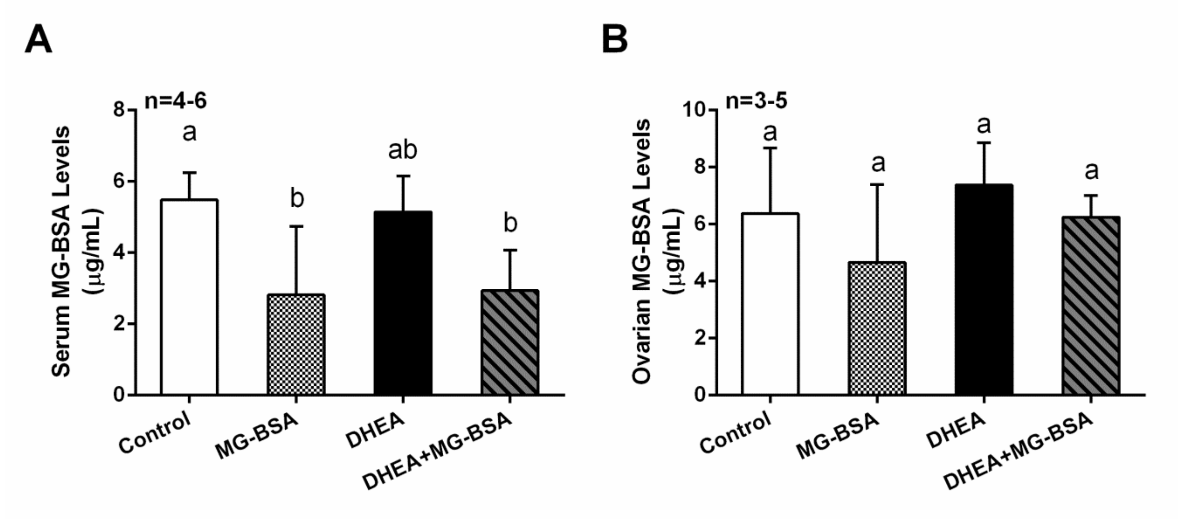

Supplementary Figure S3. The MG-BSA level in the serum and ovarian tissues. The levels of MG-BSA in **(A)** serum and **(B)** ovarian tissues were measured using MG-BSA ELISA (#STA-306; Cell Biolabs. Inc., San Diego, CA, USA). All procedures were performed according to the standard manufacturer's protocols. Data are shown as the mean ± SD.
